# The EARN-Health Trial: protocol for a randomised controlled trial to identify health effects of a financial savings programme among low-income US adults

**DOI:** 10.1136/bmjopen-2015-009366

**Published:** 2015-10-06

**Authors:** Sanjay Basu, Rita Hamad, Justin S White, Sepideh Modrek, David H Rehkopf, Mark R Cullen

**Affiliations:** Department of Medicine, Stanford University, Stanford, California, USA

**Keywords:** SOCIAL MEDICINE, PUBLIC HEALTH, MENTAL HEALTH, HEALTH ECONOMICS

## Abstract

**Introduction:**

A theory within the social epidemiology field is that financial stress related to having inadequate financial savings may contribute to psychological stress, poor mental health and poor health-related behaviours among low-income US adults. Our objective is to test whether an intervention that encourages financial savings among low-income US adults improves health behaviours and mental health.

**Methods and analysis:**

A parallel group two-arm controlled superiority trial will be performed in which 700 participants will be randomised to the intervention or a wait list. The intervention arm will be provided an online Individual Development Account (IDA) for 6 months, during which participants receive a $5 incentive (£3.2, €4.5) for every month they save $20 in their account (£12.8, €18), and an additional $5 if they save $20 for two consecutive months. Both groups will be provided links to standard online financial counselling materials. Online surveys in months 0 (prior to randomisation), 6 and 12 (6 months postintervention) will assess self-reported health behaviours and mental health among participants in both arms. The surveys items were tested previously in the US Centers for Disease Control and Prevention national health interviews and related health studies, including self-reported overall health, health-related quality of life, alcohol and tobacco use, depression symptoms, financial stress, optimism and locus of control, and spending and savings behaviours. Trial data will be analysed on an intent-to-treat basis.

**Ethics and dissemination:**

This protocol was approved by the Institutional Review Board of Stanford University (Protocol ID: 30641). The findings of the trial will be disseminated through peer-reviewed publication.

**Trial registration number:**

Identifier NCT02185612; Pre-results.

**Strengths and limitations of this study**

- Prior observational evidence provides little guidance on the impact of directly addressing specific factors linking inadequate financial savings to poor health.
- The randomised controlled nature of our study will assist in better elucidating the causal pathways from financial savings to health improvement, which are not possible to infer from observational data alone.
- The online nature of the intervention may limit its generalisability among low-income populations.
- Our study assesses the psychological effects of savings behaviour on health through the hypothesised mediator of financial stress; the study does not test the effects of increased wealth or income on health, because the intervention generates only a modest amount of savings over the short time period of the study.
- This trial uses self-reported health metrics; future studies should incorporate objective assessments if suggestive findings are observed in this initial study.

## Introduction

Extensive epidemiological research has sought to understand, and identify strategies to mitigate, the relationship between poverty and poor health.^1–8^ One strain of research on poverty and poor health has investigated the link between financial stress and psychological stress; financial stress refers to the condition of having inadequate savings or assets to pay for major expenses.^9–14^

One proposed mechanism is that financial stress leads to desperation, anxiety and hopelessness, thereby worsening overall mental health and increasing the risk that individuals will engage in tobacco smoking to obtain short-term stress relief, given a fatalistic sense of their long-term quality of life.^15^ ^16^ Consistent with this theory, recent epidemiological studies have observed that low-income individuals in the USA are at increased risk of manifesting depression symptoms, initiating tobacco smoking or binge drinking alcohol shortly after they experience financial stress associated with having inadequate savings to pay for their expenses.^17–21^ Careful ethnographic studies among low-income US adults have attributed financial stress and associated psychological stress to unemployment or inadequate income, as well as to difficulties in saving income—for example, due to numerous opportunities for low-income populations to spend earned income immediately and few opportunities to save earned income (eg, due to predatory sales and lending institutions).^22–25^ Lack of long-term savings is thought to contribute to financial stress, psychological stress and associated poor health behaviours and poor mental health even among steadily employed low-income Americans.^9^ ^11^

Research in the field of behavioural economics has lent additional insights into how inadequate savings may contribute to health-related decision-making. Recent experiments suggest that when individuals have low financial savings or limited assets to trade for money, they make more errors on cognitive performance tests and privilege short-term over long-term goals, compared with times when those same individuals experience high financial savings or assets.^26–28^ The leading theory posed to explain these observations is that having a limited financial ‘buffer’ creates desperation and focuses attention on immediate goals, to the detriment of having little mental ‘bandwidth’ left to make good decisions about the long-term.^28^ ^29^ Hence, having inadequate financial savings may pose a tremendous psychological and cognitive burden, potentially increasing the risk of making poor short-term-focused health-related decisions such as those related to tobacco smoking or excessive use of alcohol.

While several studies have investigated the health effects of strategies to increase income among US adults,^30–38^ none, to the best of our knowledge, have investigated the health effects of encouraging financial savings after income is generated. The existing literature on the health effects of income-generating interventions in the USA has been surprisingly mixed.^39^ Many observational studies have investigated the health effects of tax credits or income-generating welfare programmes affecting low-income US households, and reported a wide mix of both better and worse health outcomes.^32^ ^34–36^ ^40^ ^41^ Several studies have observed an increase in drug use, psychiatric hospitalisations and other adverse health outcomes following short-term increases in income among low-income US adults, contrary to the hypothesis that income generation would mitigate poverty's association with poor health.^42–47^

An alternative hypothesis we pose to explain these mixed findings is that income generation alone is not always sufficient to mitigate the adverse effects of poverty on health, particularly given the factor of financial stress; we hypothesise that interventions incentivising the creation of a long-term plan for financial well-being as income is generated, and assisting with the saving of income to create a financial buffer against financial stress, could help mitigate the relationship between poverty and poor health as low-income US adults generate income. To test this theory, we have planned a randomised trial, in which we will randomise low-income US adults who are earning income to an online financial savings intervention. We will compare health behaviours and mental health outcomes among individuals who receive the intervention, versus participants randomised to a wait list necessitated by limited programme capacity. The savings intervention is a type of Individual Development Account, or IDA, which is a financial intervention designed to overcome several barriers to building financial savings among low-income US adults. These barriers include financial transaction costs and cognitive burdens associated with poverty (ie, the ‘bandwidth’ issues described above) that inhibit long-term planning.^28^ ^48^ ^49^ IDAs are usually temporary savings accounts in which low-income participants can save part of their income without fees in order to build personal savings and often meet a chosen goal such as paying off credit card debt, and receive incentives to reward their savings behaviour.^49^ IDAs are typically run by registered non-profit organisations, and are offered to individuals below a threshold income level (typically 50% of county median income).^49^

A small literature has explored the economic impacts of IDAs. A retrospective uncontrolled analysis based on bank account statements from IDA participants reported that while savings outcomes vary among participants, no commonly collected demographic information (such as low income or participation in public assistance programmes) was significantly predictive of success or failure of participants in IDA programmes.^49^ Accompanying IDAs with support services such as financial counselling, rather than only having a stand-alone web platform for the IDA, was correlated to higher savings. A longitudinal study using a convenience sample of IDA participants and a matched sample of non-participants also found that participants improved their credit history and score as compared with non-participants but did not track additional outcomes.^50^ A similar study that followed participants from 14 IDA programmes across the USA for 2 years after their programme participation found that about half of the participants continued to save income after the programme, and typically used their savings for home purchases, microenterprise or postsecondary education.^51^ A subsequent controlled experiment found similar results with regard to the outcome of homeownership, but also found that many participants withdrew all funds prior to the completion of the IDA programme and did not continue saving when they lacked immediate incentives and/or a penalty for early withdrawal.^52^ This finding is concordant with the larger literature on financial incentives for health behaviour change. Studies of financial incentives for behaviour change have found that the most effective incentive strategies for changing health behaviour include provision of salient short-term incentives rather than only long-term benefits, and framing of the incentives in terms of ‘loss aversion’, which means the potential to lose future benefits.^53^ ^54^ A systematic review of the use of incentives for health behaviour change reported that incentivised programmes are typically superior to unincentivised behaviour change programmes across a broad domain of health behaviours, from tobacco smoking to physical activity engagement.^55^

Overall, participants in prior IDA studies reported reduced financial stress, increased sense of control and increased optimism, but no study, to the best of our knowledge, has evaluated the impact of the programmes on health behaviours and mental health.^49–52^ ^56^ ^57^ The rationale for our proposed study is to address this evidence gap. Our research will test the hypothesis that an IDA intervention will be superior to standard financial education in reducing unhealthy behaviours of tobacco smoking and excessive use of alcohol as well as depression symptoms. We will test this hypothesis through a parallel group randomised trial by allocating eligible low-income adults in a 1:1 ratio to a 6-month IDA intervention and 6-month post-IDA follow-up period versus a 12-month wait list control group receiving usual online financial counselling materials.^58^ The control arm is intended to simulate the resources that individuals in the intervention arm would have access to in the absence of the IDA intervention. By comparing baseline, 6 months and 12 months surveys between the two arms, we aim to identify both immediate postintervention and longer term differences between the groups in self-reported health-related quality of life, tobacco use, alcohol use and depression symptoms, as well as hypothesised mediators including financial stress, locus of control and optimism.

## Methods

A parallel group two-arm controlled superiority trial will be performed.

### Study setting

The study is a web-based experiment, taking place entirely through online activities.

### Eligibility criteria

Study participants must be English-speaking US residents aged 18 years and older who are below 50% of area median income defined by county of mailing address and household size. For example, as of June 2015, 50% of area median income would be $41 500 per year for a family of four in Los Angeles, California (£26 560, €37 350).^59^ Participants must have some earned income and must have filed taxes in the fiscal year prior to the study to confirm income-based eligibility. They must have access to the internet via computer, tablet or mobile device (eg, smartphone); have a valid email address; have a bank or credit union account; and have a valid Social Security Number or individual taxpayer ID number. Participants cannot be simultaneously enrolled in another IDA programme.

### Interventions

**Figure 1:**
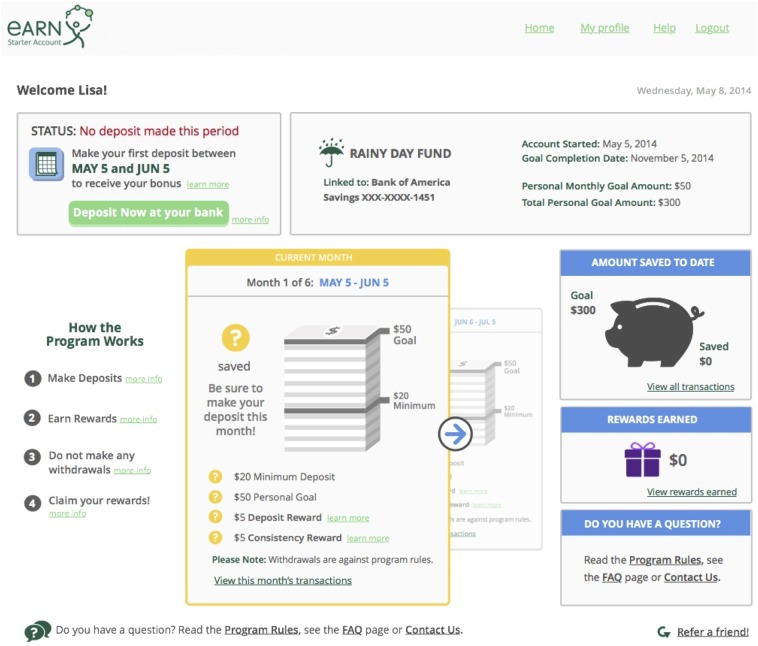
Example of a hypothetical user account web page.

The study includes two arms: The intervention arm will receive a 6-month IDA provided by the Earned Asset Resource Network (EARN), a 501(c)3 non-profit organisation based in San Francisco, California. The study investigators are unaffiliated financially or personally with EARN. EARN is among the largest providers of IDAs in the USA, having provided IDA services to approximately 5200 individuals between 2002 and 2015.^60^ EARN runs a free online service in which users who qualify for the IDA receive a 6-month personal, secure, encrypted online IDA that links to existing bank or credit union accounts. Figure 1 depicts a hypothetical user account page. Users can electronically deposit money into the IDA at any interval during the 6-month period. For every $20 a user deposits during a calendar month (£12.8, €18), EARN provides a $5 contribution into the user's IDA as an incentive to save (£3.2, €4.5). For every $20 a user deposits for two consecutive months, EARN provides an additional $5 contribution to their IDA to reward sustained savings habits (ie, providing a maximum contribution of $10 per month or $55 throughout the course of intervention). Users are asked to define a savings goal, such as saving $300 in 6 months to pay for educational expenses (£192, €270). At the end of month 6, the total savings and incentive contributions are deposited into the user's bank account and the intervention ends. Criteria for discontinuing an individual's participation are: self-reported participation in another IDA programme, early withdrawal of funds from the IDA programme, or evidence of under-reported income.

The control arm will receive notification that they are on a 12-month wait list to participate in the IDA, and will be directed to a standard US government website designed for low-literacy readers, which provides financial counselling information about how to save income.^58^ Criteria for discontinuing an individual's participation in the control arm are: self-reported participation in another IDA programme, or evidence of under-reported income.

Adherence to all intervention protocols will be enhanced by automating the process of enrolment, randomisation, notification, participation and assessment through a streamlined set of sequentially linked web pages, as detailed below.

### Outcomes

Outcomes for both arms of the trial will be assessed at months 0, 6 and 12 to identify changes from baseline in each of the outcome variables. The outcomes will be assessed through an online survey, which the individual will be prompted to complete after signing the online consent form at month 0. Participants will be prompted to complete the survey through email and telephone reminders from EARN staff; among intervention arm participants, a reminder will also appear when participants log in to their online account in months 6 and 12. All participants will receive a $20 grocery store gift card for each completed survey (£12.8, €18), distributed by postal mail to avoid interfering with the online incentive.

The complete survey text is provided in the online supplementary appendix. The primary outcome variables included in the survey are self-reported health-related quality of life; physical and mental health; tobacco and alcohol use; and depression symptoms. Self-reported health-related quality of life and physical and mental health questions are drawn from the US Centers for Disease Control and Prevention (CDC) annual telephone-based Behavioral Risk Factor Surveillance Study (BRFSS),^61^ which asks participants to compare their health with that of other people their age, and list the number of days during the past month in which their physical or mental health was not good or kept them from participating in usual activities. Tobacco and alcohol use questions were also adapted from the BRFSS and included a history of smoking, current smoking frequency and quit attempts, as well as any alcohol consumption (yes/no), number of drinks consumed during drinking days, and number of drinking episodes meeting sex-specific criteria for binge drinking. Depression symptoms were assessed using the Personal Health Questionnaire Depression Scale (PHQ-8), which includes questions such as degree of hopelessness in the past 2 weeks, measured on a Likert scale ranging from ‘not at all’ to ‘nearly every day.’

The secondary outcomes of the trial include the hypothesised mediators between the IDA intervention and health, including self-reported financial stress, locus of control and optimism. The financial stress questions are based on prior epidemiological studies relating financial stress to tobacco smoking initiation and maintenance,^62^ and include questions such as whether the participant has enough money saved to cover expenses for 3 months without any earned income (yes/no), and what their strategy is for handling a financial emergency (where response options include borrowing from a payday lender or using a credit card). The locus of control and optimism questions were adapted from the US Health and Retirement Study,^63^ and include questions about whether participants feel unable to solve problems in their lives, feel helpless or feel that good rather than bad things are more likely to happen to them; participants can respond on a six-point Likert scale from ‘strongly agree’ to ‘strongly disagree.’

Tertiary outcomes in the survey include self-reported weight and height, as obesity is related to poverty and indebtedness through possible stress and food access and affordability pathways, but is not expected to change substantially during the short course of the trial (ie, a negative control condition). Additional questions test whether the IDA produces expected improvements in long-term financial outlooks, as assessed through questions about the time course over which the participant plans for future expenses.

### Participant timeline

Table 1 provides the schedule for enrolment, intervention and assessments throughout the trial. The enrolment, intervention and assessment processes are automated through a sequence of streamlined web pages. First, interested participants arrive on a ‘landing page’ in which they provide their first and last name, email address and a password. After creating this password and confirming their email address through a hyperlink sent to their email, users provide their mailing address, demographic information (date of birth, sex, race/ethnicity, marital status, education completed, employment status, household size and annual household income) and Social Security or individual tax ID number. They also answer key qualifying information regarding whether they have a bank account and file taxes, and they upload their 1040 tax form from the prior tax year to verify income. Participants who do not qualify for the eligibility criteria are informed of their denial; those who qualify next complete an informed consent page (reproduced in the online supplementary appendix). If they consent to participate, they then receive the month 0 survey. After the month 0 survey, they are randomised to the intervention or control arm.

**Table 1:** Schedule of enrolment, interventions and assessments

Time point	Study period				
	Enrolment	Allocation	Postallocation		Close-out
	-t_1_	0	t_6_	t_12_	t_12_
Enrolment					
Eligibility screen	X				
Informed consent	X				
Allocation		X			
Interventions					
IDA		X	X	X	X
Wait list control		X	X	X	X
Assessments					
Online survey		X	X	X	
Process evaluation interviews					X

IDA, Individual Development Account.

Next, intervention arm participants are shown an information page about how to use the IDA and are asked to link to their bank account at any US Federal Deposit Insurance Corporation (FDIC)-insured bank or National Credit Union Association (NCUA)-insured credit union through a secure, encrypted linkage system similar to that used by employers for direct deposit transactions. Participants without a bank or credit union account are provided a list of local bank and credit union branches and informed that they should look for an account with no minimum balance requirements, no monthly maintenance fees, and FDIC or NCUA insurance; they are able to return to the website after setting up an account. Participants then see their personalised IDA home page shown in figure 1. The control group receives a notice that they are on a 12-month wait list, is notified to fill out a survey every 6 months throughout the wait list period for a $20 grocery store gift card (£12.8, €18) as long as they do not participate in another IDA service, and receives a hyperlink to standard online financial advice.^58^

Participants in both arms then receive email and telephone notifications to complete surveys in months 6 and 12. The intervention arm also receives the survey after login. The $20 grocery store gift cards are mailed by research staff to participants in the week following their survey completion. The intervention arm participants will close-out of the intervention and have their incentives deposited to their bank account at month 6. As per the EARN IDA rules, participants who withdraw from the programme before 6 months only receive their savings but not their incentives, as they are notified during the enrolment process. Both the saving and incentive earnings over 6 months are deposited at the 6-month time point into their bank account, but not before. Following the intervention, participants will be required to be free of any IDA participation, similar to the control group, for an additional 6 months after completion of the month 12 survey in order to compare longer term outcomes between the two groups. Close-out of the study will occur after completion of all month 12 surveys or non-response by a participant for 1 month following the month 12 email and telephone notification. Participants in the wait-listed group will be informed that they will be offered the intervention promptly after the 12-month survey. As per EARN's policy, participants in the intervention group will not be allowed to reuse an EARN IDA in the future, as the intervention is a 6-month, one-time activity for all individuals, as is typical of IDAs.^49^

### Sample size

As there is no prior literature estimating the impact of IDAs on the studied health outcomes, we powered the study conservatively without a priori information to estimate the potential effect size. Taking into account three repeated surveys per person and assuming a typical correlation of 0.5 among the repeated measures, we would need 526 participants distributed equally between the two arms to detect a small effect size difference of Cohen's f=0.1 (for analysis of variance (ANOVA) analysis^64^) between the two groups with 80% power at an α error probability of 0.05 (G*Power software V.3.1). Conservatively assuming 25% attrition, we have aimed for a sample size of N=700.

**Figure 2:**
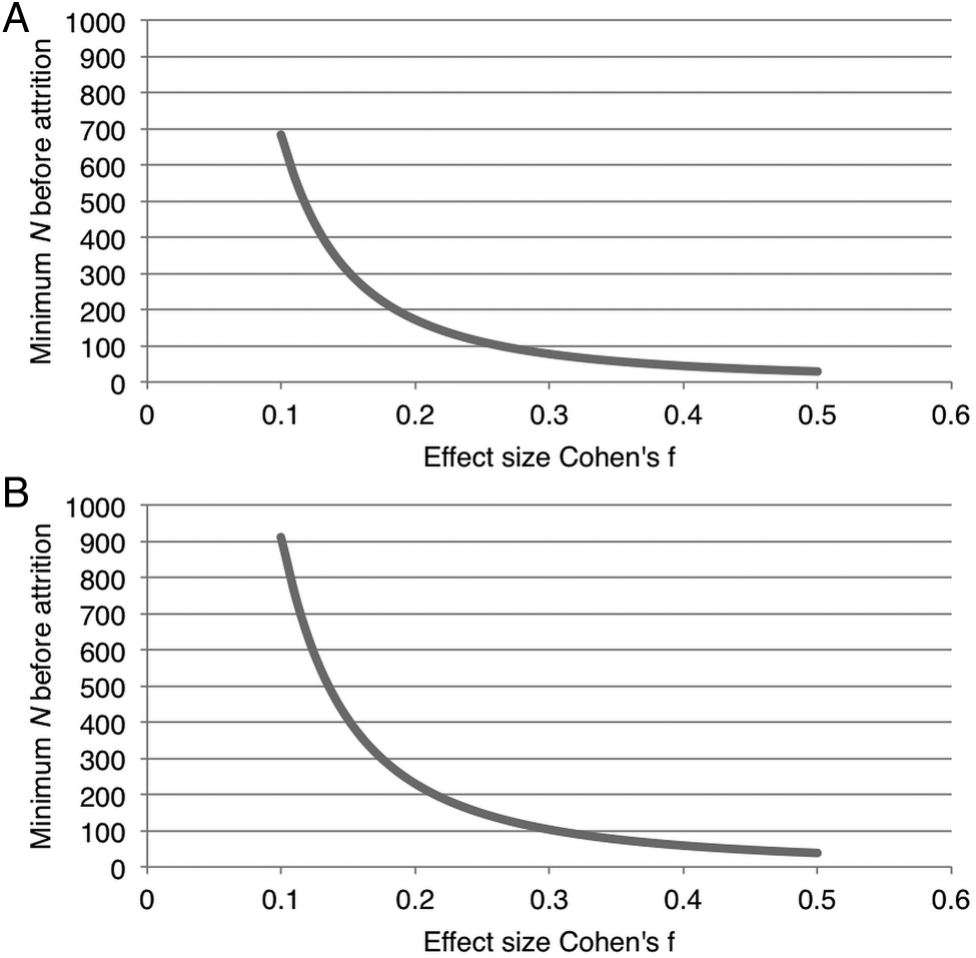
The study was powered to detect a conservative effect size of Cohen's f=0.1 with 80% power at an α error probability of 0.05. The sample size was calculated assuming two study arms, three repeated surveys (months 0, 6, and 12) for each participant, a 25% attrition rate, and a correlation of 0.5 among repeated measures. We display sensitivity analysis graphs of the preattrition minimum sample size needed to detect the estimated effect size, using G*Power software (V.3.1). The graphs display the minimal sample size required under alternative effect size estimates besides f=0.1—(A) when increasing the assumed correlation between repeated measures from 0.5 to 0.8 and (B) when attempting to achieve a power of 0.9 rather than 0.8.

We conducted further sensitivity analyses around this sample size to examine how differences in effect size would alter power for the design; these are plotted in figure 2. These plots demonstrate that the necessary sample size before attrition drops to less than 250 with an effect size of f=0.15, while at least 90% power would be achieved with our chosen sample size and assumed attrition rate if the effect size were f=0.12 or larger. As also shown in figure 2, the effect size would have to be at least f=0.13 to have 80% power with our chosen sample size if the correlation between repeated measures is as high as 0.8.

### Recruitment

We anticipate that recruitment will require 3 months to achieve the targeted sample size. Our strategies to achieve adequate participant enrolment during these months are to advertise the study at local community colleges whose enrollees share the typical demographics of IDA users; advertise in local newspapers, radio and online news services; and recruit from a network of local primary care medical clinics serving low-income populations in the greater San Francisco Bay Area. Although geographic location is not an explicit criterion for inclusion or exclusion, many EARN users live in the San Francisco Bay Area where EARN's headquarters are located.

### Allocation

Computer-generated random numbers will be used to generate the allocation sequence, with no factors for stratification and no planned restrictions (eg, blocking). The online system will be programmed to automatically generate these numbers such that the sequence will remain concealed until interventions are assigned automatically by the website. To preserve blinding among all data analysts, staff at EARN will code the survey response data such that the intervention arm will be coded numerically and the numerical key will be blinded from the research data team including all data analysts. Unblinding will not be permitted to any data analyst during the trial. EARN staff who are not involved in data analysis will be unblinded to ensure that they can respond to email or phone queries from participants who have questions about the logistics of using the IDA page or need assistance entering information to link their online account to their bank account. These correspondences will not involve the research data analysts, and a strict information restriction protocol will prevent EARN staff from disclosing any participant allocation details to research staff including research data analysts throughout the trial and post-trial analysis.

### Data collection methods

Collection of demographic and survey data will be entirely online, with EARN staff screening for duplicate accounts to prevent threats to data quality. Participants will receive gift cards only after submission of each survey, to reduce the likelihood of missing data or blank surveys. To promote retention and complete follow-up, EARN staff will call participants who have not completed a survey 1 week after automated email notification.

The consistency and validity of the survey measures among low-income US adults have been published previously as detailed below. For the overall self-rated health questions, internal consistency is satisfactory (Cronbach's α>0.78) and criterion validity was moderate-to-high as assessed by correlations with physical health scales (ρ=0.86, p<0.0001). Known-groups validity was also significant at an α level of 0.05 when comparing self-reported health between people with and without medical diagnoses indicative of chronic disease.^65^ For the tobacco and alcohol survey items, high internal consistency has been reported (Cronbach's α>0.85),^66^ as well as high validity assessed through comparison of population prevalence comparisons across surveys using the items versus direct measurement in national surveys (<1% absolute prevalence difference).^67–71^ The financial stress questions have satisfactory internal consistency (α>0.67)^72^ and are significantly associated with confirmed earnings, savings and expenditures.^73^ The PHQ survey items are used in clinical practice as a screening instrument for depression, having satisfactory internal consistency and high validity (Cronbach's α=0.82 and goodness-of-fit index of 0.98 on confirmatory factor analysis of emotional and somatic dimensions of depression symptoms among adults).^74^ The optimism questions have satisfactory internal consistency (Cronbach's α>0.78) and significant predictive and discriminant validity for depression and coping success when taking account effects of neuroticism, trait anxiety, self-mastery and self-esteem in a sample of adults.^75–77^ The locus of control questions has a satisfactory internal consistency (Cronbach's α>0.86) and high validity for predicting life satisfaction and depression symptoms.^78–80^

### Data management

Data entry is automated from the survey website and saved on a secure, encrypted server that meets Health Information Portability and Accountability Act (HIPAA) standards. The survey results are coded with a participant ID rather than a name or identifying information, and the study arm is similarly coded (1/2) to preserve blinding of data analysts. Data analysts will check for any double data entries/duplicate surveys by screening participant ID numbers for duplicated surveys at the same time point, keeping only the survey responses that correspond to the later/completed survey in case an individual experienced website reloading and survey resetting. Range checks will be performed on all data values to ensure no errors in the survey codes. The online survey has been tested to ensure correct capture of entered survey responses.

### Statistical methods and data analysis plan

We plan an intent-to-treat analysis using mixed models to compare survey responses between the two groups, with demographic covariates (age, sex, race/ethnicity and income) and individual random effects.^81^ For i=1,…, N participants with j=1,…,n_i_ repeated observations nested within each participant, we will specify a random participant intercept and random linear time-trend model, n_ij_=**x**_ij_β+**z**_ij_v_i_, where **x** is the vector of regressors with coefficients β, **z** a vector of variables having random effects, and v the random effects coefficients. A link function g(·) is specified which converts the expected value μ_ij_ of the outcome variable, Y (ie, μ_ij_=E[Y_ij_|v_i_,**x**_ij_]), to predictor n_ij_. We plan to use logit link functions for binary outcomes, ordered logit link functions for Likert scale outcomes, and identity or log links for continuous outcome variables depending on data skewness. Robust SEs will be used to calculate 95% CIs, and a maximum likelihood procedure will be adopted for model fitting. Owing to the likelihood of a higher dropout among the wait-listed participants, survivorship bias will be tested by comparing baseline demographic characteristics between arms. Participants lost to follow-up will be censored at the time of the last survey they completed and included in the analysis, with missing data handled using likelihood-based pattern mixture analysis, which has been found to be superior in detecting and correcting for bias as compared with the Last Observation Carried Forward method or multiple imputation with chained equations when missingness is potentially informative.^82–85^

### Process evaluation

To assist in the interpretation of the quantitative results, participants in both trial arms will be asked if they are willing to engage in a brief, anonymous telephone interview to further elicit qualitative responses to the intervention for an additional $10 gift card (£6.4, €9). Those participants who consent to participate in telephone interviews will be called by research staff after the quantitative data analysis is complete, and answer open-ended questions to further understand the linkages between the intervention and self-reported health outcomes. The conversations will focus on whether the participants received the incentives and found them to be helpful prompts to engage in savings; whether the participants were engaging in other savings programmes or any health programmes that may have affected their survey responses; how they felt about the programme rules and the duration of the intervention; whether they have suggestions for intervention refinement; and (in the control group) whether the participants enrolled in alternative savings programmes or read the online material that was provided as an alternative to the IDA intervention.

### Data monitoring

A data monitoring committee will consist of external scientists with expertise on financial stress and health behaviour, including a chairperson (Arjumand Siddiqi, University of North Carolina Chapel Hill/University of Toronto) and an independent trial steering committee (Aaron Reeves, University of Oxford; Mauricio Avendano-Pabon, Harvard University/London School of Economics; Adam Coutts, London School of Hygiene and Tropical Medicine). Its role is to monitor progress, ensure fidelity and accurate collection of data by identifying any reasons for duplicate or erroneous survey data responses and reporting to the overall group of investigators (coauthors of this manuscript), and monitoring any safety or ethics concerns that arise during the trial. The data monitoring committee is independent from the sponsor of the trial. No interim analyses will be performed, and no trial discontinuation criteria have been formulated as it is not anticipated that the trial would need to stop prematurely given the minimal risks to participants.

### Harms

Adverse events are likely to be restricted to the risk of loss of confidentiality, and psychological stress from completion of surveys that involve potentially sensitive health information. To minimise potential harms, loss of confidentiality will be minimised by using only password-protected encrypted servers for data storage, with personal information protections detailed further below. To reduce psychological harms, the principal investigator's contact information is provided with the consent form, and contact information for EARN staff is provided with surveys and on the IDA website, to allow referral of distressed participants to area mental health services or medical providers if needed. All adverse events will be reported to the Stanford University Institutional Review Board.

### Auditing

No specific auditing has been planned for external supervision of trial conduct.

## Ethics and dissemination

### Protocol amendments

Protocol modifications including changes to eligibility criteria, outcomes or analyses will be reported to the Institutional Review Board of Stanford University and posted on the clinicaltrials.gov registry.

### Consent

Informed consent will be obtained through an online web page that appears before study enrolment, and replicated in the online supplementary appendix.

### Confidentiality

Personal information about potential and enrolled participants will be collected during the enrolment process through EARN's secure, encrypted online system. The personal information will be held on the secure server and not shared before, during or after the trial. The personal information will be used to mail participants their $20 gift card reward for survey completion, and place telephone calls and email reminders for surveys. Subsequently, the information will be deleted. Trial data will be maintained after the trial using only a participant ID, the study arm code and the de-identified survey results for months 0, 6 and 12.

### Ancillary and post-trial care

Ancillary and post-trial care is not deemed likely for any participants from this study. Those participants who suffer psychological harm during the course of the study will be referred to area medical care services.

### Dissemination policy

The investigators plan to communicate trial results in a peer-reviewed publication and potentially at academic public health conferences. No restrictions on publication exist. Authorship will follow the International Council of Medical Journal Editors standards for authorship, and no professional writers will be used.^86^ The full protocol will be accessible via clinicaltrials.gov. Participant-level data and statistical code will be available on request for non-commercial academic research purposes.

Three additional modes of dissemination will be employed. First, we will present the results to an annual gathering of participants in EARN IDA programmes, to elicit responses from participants for potential future IDA programme refinement and additional health pathways for potential investigation. Second, we will present the results to the National Coordinating Center for Public Health Services and Systems Research, which is a consortium of public health departments around the country who are part of a consortium addressing social and economic determinants of health through their local communities. Finally, the study results will be presented at the Stanford Center on Longevity's annual conference on financial security, which is attended by the major financial institutions seeking to fund philanthropic ventures that improve both financial security and quality of life across the life course.

## References

[1] Marmot M, Friel S, Bell R Closing the gap in a generation: health equity through action on the social determinants of health. Lancet 2008;372:1661–9. 10.1016/S0140-6736(08)61690-6 18994664

[2] Marmot M Social determinants of health inequalities. Lancet 2005;365:1099–104. 10.1016/S0140-6736(05)71146-6 15781105

[3] Marmot M, Wilkinson R Social determinants of health. Oxford University Press, 2005 https://books.google.com/books?hl=en&lr=&id=AmwiS8HZeRIC&oi=fnd&pg=PA17&dq=who+social+determinants&ots=y_FB74SmJ_&sig=ZVomjfirgHLJr-DyAUhrEb28Vqg (accessed 25 Jun 2015).

[4] Flint AJ, Novotny TE Poverty status and cigarette smoking prevalence and cessation in the United States, 1983–1993: the independent risk of being poor. Tob Control 1997;6:14–18. 10.1136/tc.6.1.14 9176981 PMC1759538

[5] Cerdá M, Diez-Roux AV, Tchetgen ET The relationship between neighborhood poverty and alcohol use: estimation by marginal structural models. Epidemiology 2010;21:482 10.1097/EDE.0b013e3181e13539 20498603 PMC3897210

[6] Khan S, Murray RP, Barnes GE A structural equation model of the effect of poverty and unemployment on alcohol abuse. Addict Behav 2002;27:405–23. 10.1016/S0306-4603(01)00181-2 12118628

[7] Belle D Poverty and women's mental health. Am Psychol 1990;45:385 10.1037/0003-066X.45.3.385

[8] Leon DA, Walt G, eds. Poverty, inequality, and health: an international perspective. Oxford University Press, 2001 http://www.cabdirect.org/abstracts/20023007902.html (accessed 24 Oct 2014).

[9] Siahpush M, Spittal M, Singh GK Smoking cessation and financial stress. J Public Health Oxf Engl 2007;29:338–42. 10.1093/pubmed/fdm070 17998258

[10] Bagwell DC, Kim J Financial stress, health status, and absenteeism in credit counseling clients. J Consum Educ 2003;20:50–8.

[11] Peirce RS, Frone MR, Russell M Financial stress, social support, and alcohol involvement: a longitudinal test of the buffering hypothesis in a general population survey. Health Psychol 1996;15:38 10.1037/0278-6133.15.1.38 8788539

[12] Siahpush M, Spittal M, Singh GK Association of smoking cessation with financial stress and material well-being: results from a prospective study of a population-based national survey. Am J Public Health 2007;97:2281–7. 10.2105/AJPH.2006.103580 17971550 PMC2089113

[13] Wrosch C, Heckhausen J, Lachman ME Primary and secondary control strategies for managing health and financial stress across adulthood. Psychol Aging 2000;15:387 10.1037/0882-7974.15.3.387 11014704

[14] Siahpush M, Carlin JB Financial stress, smoking cessation and relapse: results from a prospective study of an Australian national sample. Addiction 2006;101:121–7. 10.1111/j.1360-0443.2005.01292.x 16393198

[15] Dorsett R, Marsh A The health trap: poverty, smoking and lone parenthood. London: Policy Studies Institute, 1998 http://www.opengrey.eu/item/display/10068/379056 (accessed 6 Jul 2015).

[16] Graham H When life's a drag: women, smoking and disadvantage. London: HM Stationery Office, 1993 http://www.opengrey.eu/item/display/10068/484501 (accessed 6 Jul 2015).

[17] Nettleton S, Burrows R Mortgage debt, insecure home ownership and health: an exploratory analysis. Sociol Health Illn 1998;20:731–53. 10.1111/1467-9566.00127

[18] Nelson MC, Lust K, Story M Credit card debt, stress and key health risk behaviors among college students. Am J Health Promot 2008;22:400–7. 10.4278/ajhp.22.6.400 18677880

[19] Balmer N, Pleasence P, Buck A Worried sick: the experience of debt problems and their relationship with health, illness and disability. Soc Policy Soc 2006;5:39–51. 10.1017/S147474640500271X

[20] Drentea P, Lavrakas PJ Over the limit: the association among health, race and debt. Soc Sci Med 2000;50:517–29. 10.1016/S0277-9536(99)00298-1 10641804

[21] Cooke R, Barkham M, Audin K Student debt and its relation to student mental health. J Furth High Educ 2004;28:53–66. 10.1080/0309877032000161814

[22] Ehrenreich B Nickel and dimed: on (not) getting by in America. Macmillan, 2010 https://books.google.com/books?hl=en&lr=&id=SfAgn7KXggIC&oi=fnd&pg=PP9&dq=nickeled+and+dimed&ots=rp93hgKpNA&sig=Ql7A11hSk8g33I75KZocFbFJEts (accessed 6 Jul 2015).

[23] Newman KS No shame in my game: the working poor in the inner city. Vintage, 2009 https://books.google.com/books?hl=en&lr=&id=1jfAhghdH7MC&oi=fnd&pg=PR9&dq=related:ge8OpW9HpVoJ:scholar.google.com/&ots=u_BtXUyngY&sig=0v8UC2vZmrkd4UNuihI2iRBDrFM (accessed 6 Jul 2015).

[24] Shipler DK The working poor: invisible in America. Vintage, 2008 https://books.google.com/books?hl=en&lr=&id=YAxIC17Bso8C&oi=fnd&pg=PR9&dq=related:ge8OpW9HpVoJ:scholar.google.com/&ots=cGNw9Vs6S5&sig=dtZIrhWhBDyuXDXQRZTwBI-1xWg (accessed 6 Jul 2015).

[25] Mishel L, Bivens J, Gould E The state of working America. Cornell University Press, 2012 https://books.google.com/books?hl=en&lr=&id=WdM77z0HUcAC&oi=fnd&pg=PR9&dq=related:ge8OpW9HpVoJ:scholar.google.com/&ots=bTndjdvV0q&sig=KVwaNneRSaRjUVlFoPTg1qGDQfc (accessed 6 Jul 2015).

[26] Mani A, Mullainathan S, Shafir E Poverty impedes cognitive function. Science 2013;341:976–80. 10.1126/science.1238041 23990553

[27] Shah AK, Mullainathan S, Shafir E Some consequences of having too little. Science 2012;338:682–5. 10.1126/science.1222426 23118192

[28] Mullainathan S, Shafir E Scarcity: why having too little means so much. New York: Times Books, 2013.

[29] Zwane AP Implications of scarcity. Science 2012;338:617–18. 10.1126/science.1230292 23118174

[30] Kehrer BH, Wolin CM Impact of income maintenance on low birth weight: evidence from the Gary Experiment. J Hum Resour 1979;14:434–62. 10.2307/145316 575154

[31] Huston AC, Duncan GJ, McLoyd VC Impacts on children of a policy to promote employment and reduce poverty for low-income parents: new hope after 5 years. Dev Psychol 2005;41:902–18. 10.1037/0012-1649.41.6.902 16351336

[32] Bitler MP, Gelbach JB, Hoynes HW Welfare reform and health. J Hum Resour 2005;40:309–34.

[33] Hoynes HW, Miller DL, Simon D Income, the earned income tax credit, and infant health. National Bureau of Economic Research, 2012 http://www.nber.org/papers/w18206 (accessed 25 Jun 2015). 23304766

[34] Bruckner TA, Rehkopf DH, Catalano RA Income gains and very low-weight birth among low-income black mothers in California. Biodemography Soc Biol 2013;59:141–56. 10.1080/19485565.2013.833802 24215256

[35] Rehkopf DH, Strully KW, Dow WH The short-term impacts of Earned Income Tax Credit disbursement on health. Int J Epidemiol 2014;43:1884–94. 10.1093/ije/dyu172 25172139 PMC4342690

[36] Strully KW, Rehkopf DH, Xuan Z Effects of prenatal poverty on infant health state earned income tax credits and birth weight. Am Sociol Rev 2010;75:534–62. 10.1177/0003122410374086 21643514 PMC3104729

[37] Herd P, Schoeni RF, House JS Upstream solutions: does the supplemental security income program reduce disability in the elderly? Milbank Q 2008;86:5–45. 10.1111/j.1468-0009.2007.00512.x 18307476 PMC2690339

[38] Williams DR, Costa MV, Odunlami AO Moving upstream: how interventions that address the social determinants of health can improve health and reduce disparities. J Public Health Manag Pract 2008;14 (Suppl):S8–17. 10.1097/01.PHH.0000338382.36695.42 18843244 PMC3431152

[39] Adda J, Banks J, Von Gaudecker H-M The impact of income shocks on health: evidence from cohort data. J Eur Econ Assoc 2009;7:1361–99. 10.1162/JEEA.2009.7.6.1361

[40] Bitler M, Hoynes HW Welfare reform and indirect impacts on health. National Bureau of Economic Research, 2006 http://www.nber.org/papers/w12642 (accessed 25 Jun 2015).

[41] Grogger J, Karoly LA, Klerman JA Consequences of welfare reform: a research synthesis. Santa Monica: The RAND Corporation, 2002 http://lbr.rand.org/pubs/drafts/DRU2676.html (accessed 24 Oct 2014).

[42] Kawachi I, Adler NE, Dow WH Money, schooling, and health: mechanisms and causal evidence. Ann N Y Acad Sci 2010;1186:56–68. 10.1111/j.1749-6632.2009.05340.x 20201868

[43] Bruckner TA, Brown RA, Margerison-Zilko C Positive income shocks and accidental deaths among Cherokee Indians: a natural experiment. Int J Epidemiol 2011;40:1083–90. 10.1093/ije/dyr073 21527447 PMC3156370

[44] Phillips DP, Christenfeld N, Ryan NM An increase in the number of deaths in the United States in the first week of the month—an association with substance abuse and other causes of death. N Engl J Med 1999;341:93–8. 10.1056/NEJM199907083410206 10395634

[45] Swartz JA, Hsieh C, Baumohl J Disability payments, drug use and representative payees: an analysis of the relationships. Addiction 2003;98:965–75. 10.1046/j.1360-0443.2003.00414.x 12814502

[46] Catalano R, McConnell W, Forster P Does the disbursement of income increase psychiatric emergencies involving drugs and alcohol? Health Serv Res 2000;35:813. 11055450 PMC1089154

[47] Samet J Relapse triggers—full moon, full wallet, or foolhardy? Am J Med 2001;110:406–7. 10.1016/S0002-9343(01)00639-8 11286959

[48] Barr MS Banking the poor: policies to bring low-income Americans into financial mainstream. Brookings Institution, Research Brief, September 2004. Published Online First: 2004. http://papers.ssrn.com/sol3/papers.cfm?abstract_id=722002 (accessed 25 Jun 2015).

[49] Schreiner M, Sherraden MW Can the poor save? Saving & asset building in Individual Development Accounts. Transaction Publishers, 2007.

[50] Birkenmaier J, Curley J, Kelly P Credit building in IDA programs early findings of a longitudinal study. Res Soc Work Pract 2012;22:605–14. 10.1177/1049731512453208

[51] Schreiner M, Clancy M, Sherraden M Saving performance in the American dream demonstration. St. Louis, MO: Center for Social Development, Washington University Published Online First: 2002. http://www.usc.edu/dept/chepa/IDApays/publications/ADDReport2002.pdf (accessed 25 Jun 2015).

[52] Mills G, Gale WG, Patterson R Effects of Individual Development Accounts on asset purchases and saving behavior: evidence from a controlled experiment. J Public Econ 2008;92:1509–30. 10.1016/j.jpubeco.2007.09.014

[53] Rogers T, Milkman KL, Volpp KG Commitment devices: using initiatives to change behavior. JAMA 2014;311:2065–6. 10.1001/jama.2014.3485 24777472

[54] Halpern SD, French B, Small DS Randomized trial of four financial-incentive programs for smoking cessation. N Engl J Med 2015;372:2108–17. 10.1056/NEJMoa1414293 25970009 PMC4471993

[55] Giles EL, Robalino S, McColl E The effectiveness of financial incentives for health behaviour change: systematic review and meta-analysis. PLoS ONE 2014;9:e90347 10.1371/journal.pone.0090347 24618584 PMC3949711

[56] Clancy M, Grinstein-Weiss M, Schreiner M Financial education and savings outcomes in Individual Development Accounts. Working Paper 01-2 St. Louis, MO: Center for Social Development, Washington University, 2001 http://microfinance.com/English/Papers/IDAs_Financial_Education.pdf (accessed 14 Nov 2014).

[57] Sherraden M From research to policy: lessons from Individual Development Accounts. J Consum Aff 2000;34:159–81. 10.1111/j.1745-6606.2000.tb00089.x

[58] USA.gov. Help for Difficult Financial Times 2015 http://www.usa.gov/citizen/topics/family/help-for-difficult-financial-times.shtml (accessed 25 Jun 2015).

[59] Housing Authority of the City of Los Angeles. FY 2015 Public Housing & Section 8 Income Limits. 2015 http://www.hacla.org/applicantinfo/ (accessed 25 Jun 2015).

[60] Earned Asset Resource Network. About EARN. 2015 http://www.earn.org/about_earn (accessed 25 Jun 2015).

[61] Centers for Disease Control and Prevention. Survey Data & Documentation. Behavioral Risk Factor Surveillance System. 2015 http://www.cdc.gov/brfss/data_documentation/index.htm (accessed 25 Jun 2015).

[62] Siahpush M, Yong H-H, Borland R Smokers with financial stress are more likely to want to quit but less likely to try or succeed: findings from the International Tobacco Control (ITC) Four Country Survey. Addiction 2009;104:1382–90. 10.1111/j.1360-0443.2009.02599.x 19438837 PMC2714876

[63] Juster FT, Suzman R An overview of the health and retirement study. J Hum Resour 1995;30:S7–56. 10.2307/146277

[64] Cohen J A power primer. Psychol Bull 1992;112:155 10.1037/0033-2909.112.1.155 19565683

[65] Horner-Johnson W, Krahn G, Andresen E Developing summary scores of health-related quality of life for a population-based survey. Public Health Rep 2009;124:103–10. 19413032 10.1177/003335490912400113 PMC2602935

[66] Patel SA, Narayan KMV, Ali MK Interstate variation in modifiable risk factors and cardiovascular mortality in the United States. PLoS ONE 2014;9:e101531 10.1371/journal.pone.0101531 25003975 PMC4086813

[67] Pierannunzi C, Hu SS, Balluz L A systematic review of publications assessing reliability and validity of the Behavioral Risk Factor Surveillance System (BRFSS), 2004–2011. BMC Med Res Methodol 2013;13:49 10.1186/1471-2288-13-49 23522349 PMC3622569

[68] Bowlin SJ, Morrill BD, Nafziger AN Validity of cardiovascular disease risk factors assessed by telephone survey: the Behavioral Risk Factor Survey. J Clin Epidemiol 1993;46:561–71. 10.1016/0895-4356(93)90129-O 8501483

[69] Brownson RC, Jackson-Thompson J, Wilkerson JC Reliability of information on chronic disease risk factors collected in the Missouri Behavioral Risk Factor Surveillance System. Epidemiology 1994;5:545–9. 7986871

[70] Stein AD, Lederman RI, Shea S The Behavioral Risk Factor Surveillance System questionnaire: its reliability in a statewide sample. Am J Public Health 1993;83:1768–72. 10.2105/AJPH.83.12.1768 8259816 PMC1694908

[71] Stein AD, Courval JM, Lederman RI Reproducibility of responses to telephone interviews: demographic predictors of discordance in risk factor status. Am J Epidemiol 1995;141:1097–105. 7771444 10.1093/oxfordjournals.aje.a117375

[72] NICHD Early Child Care Research Network. Child care and child development: results from the NICHD study of early child care and youth development. Guilford Press, 2005.

[73] Grable JE, Archuleta K, Roy RN Financial planning and counseling scales. Springer Science & Business Media, 2010.

[74] Pressler SJ, Subramanian U, Perkins SM Measuring depressive symptoms in heart failure: validity and reliability of the patient health questionnaire-8. Am J Crit Care 2011;20:146–52. 10.4037/ajcc2010931 20378777

[75] Scheier MF, Carver CS, Bridges MW Distinguishing optimism from neuroticism (and trait anxiety, self-mastery, and self-esteem): a reevaluation of the Life Orientation Test. J Pers Soc Psychol 1994;67:1063–78. 10.1037/0022-3514.67.6.1063 7815302

[76] Taylor SE, Kemeny ME, Reed GM Psychological resources, positive illusions, and health. Am Psychol 2000;55:99–109. 10.1037/0003-066X.55.1.99 11392870

[77] Peterson C The future of optimism. Am Psychol 2000;55:44–55. 10.1037/0003-066X.55.1.44 11392864

[78] Lachman ME, Weaver SL The sense of control as a moderator of social class differences in health and well-being. J Pers Soc Psychol 1998;74:763–73. 10.1037/0022-3514.74.3.763 9523418

[79] Pearlin LI, Schooler C The structure of coping. J Health Soc Behav 1978;19:2–21. 10.2307/2136319 649936

[80] Hampson SE Personality processes: mechanisms by which personality traits ‘get outside the skin’. Annu Rev Psychol 2012;63:315–39. 10.1146/annurev-psych-120710-100419 21740225 PMC3193854

[81] McCullagh P, Nelder JA Generalized linear models. CRC Press, 1989 https://books.google.com/books?hl=en&lr=&id=h9kFH2_FfBkC&oi=fnd&pg=PR16&dq=generalized+linear+models&ots=JhRY7VQNvN&sig=kzIm5DN8mUT4I6XGTtTcWSZDc6w (accessed 25 Jun 2015).

[82] Mallinckrodt CH, Clark WS, David SR Type I error rates from mixed effects model repeated measures versus fixed effects ANOVA with missing values imputed via last observation carried forward. Drug Inf J 2001;35:1215–25. 10.1177/009286150103500418

[83] Siddiqui O, Ali MW A comparison of the random-effects pattern mixture model with last-observation-carried-forward (LOCF) analysis in longitudinal clinical trials with dropouts. J Biopharm Stat 1998;8:545–63. 10.1080/10543409808835259 9855033

[84] Hamer RM, Simpson PM Last observation carried forward versus mixed models in the analysis of psychiatric clinical trials. Am J Psychiatry 2009;166:639–41. 10.1176/appi.ajp.2009.09040458 19487398

[85] Schafer J Missing data in longitudinal studies: a review. American Association of Psychological Science, Nashville, 2005.

[86] International Committee of Medical Journal Editors. Uniform requirements for manuscripts submitted to biomedical journals. Pathology (Phila) 1997;29:441–7.

